# Burden of syphilis in Brazil and federated units, 1990-2016: estimates from the Global Burden of Disease Study 2019

**DOI:** 10.1590/0037-8682-0010-2022

**Published:** 2022-07-25

**Authors:** Juliana Maria Trindade Bezerra, Pedro Alves Soares Vaz de Castro, Carla Jorge Machado, Mariângela Carneiro

**Affiliations:** 1 Universidade Federal de Minas Gerais, Instituto de Ciências Biológicas, Departamento de Parasitologia, Programa de Pós-Graduação em Parasitologia, Belo Horizonte, MG, Brasil.; 2 Universidade Estadual do Maranhão, Centro de Ciências Agrárias, Programa de Pós-Graduação em Ciência Animal, São Luís, MA, Brasil.; 3 Universidade Estadual do Maranhão, Centro de Estudos Superiores de Lago da Pedra, Curso de Licenciatura em Ciências Biológicas, Lago da Pedra, MA, Brasil.; 4 Universidade Federal de Minas Gerais, Faculdade de Medicina, Curso de Bacharelado em Medicina, Belo Horizonte, MG, Brasil.; 5 Universidade Federal de Minas Gerais, Faculdade de Medicina, Programa de Programa de Pós-Graduação em Promoção de Saúde e Prevenção da Violência, Belo Horizonte, MG, Brasil.; 6 Universidade Federal de Minas Gerais, Faculdade de Medicina, Programa de Pós-Graduação em Infectologia e Medicina Tropical, Belo Horizonte, MG, Brasil.; 7 Universidade Federal de Ouro Preto, Núcleo de Pesquisas em Ciências Biológicas, Programa de Pós-Graduação em Ciências Biológicas, Ouro Preto, MG, Brasil.

**Keywords:** Syphilis, DALYs, YLLs, YLDs, Population estimates, Burden disease metrics

## Abstract

**Background:**

Syphilis is a chronic infectious disease that has created challenging situations for humanity for centuries. Transmission can occur sexually or vertically, with great repercussions on populations, particularly among women and children. The present study presents information on the main burden imposed by syphilis generated by the Global Burden of Disease (GBD) Study 2019 for Brazil and its 27 federated units.

**Methods:**

We described the metrics of incidence, deaths, years of life lost (YLLs), years lived with disability (YLDs), and disability-adjusted life years (DALYs), standardized by age and per 100,000 inhabitants, from 1990 to 2019, and we compared the disease burden between the years 1990 and 2019.

**Results:**

In Brazil, the disease burden increased between 2005 and 2019 for all metrics. Although a higher incidence of syphilis was found among women in 2019, DALYs [YLLs (males: 15.9%; females: 21.8%), YLDs (males: 25.0%; females: 50.0%), and DALYs (males: 16.2%; females: 22.4%)] were higher among men. In 2019, the highest DALY rate per 100,000 inhabitants was observed in individuals aged above 50 years. The State of Maranhão presented the highest values of DALYs {1990: 165.2 [95% uncertainty interval (UI) 96.2-264.4]; 2005: 43.8 [95% UI 30.3-62.4]; 2019: 29.1 [95% UI 19.8-41.1]} per 100,000 inhabitants in the three years analyzed.

**Conclusions:**

The burden of syphilis has increased in recent years. Men presented higher DALYs, although the incidence of the disease was higher in women. Syphilis affects a large number of people across all age groups, causing different degrees of disability and premature death (DALYs).

## INTRODUCTION

Syphilis is an important infectious disease caused by *Treponema pallidum*, a spirochete first identified in 1905^1^. This etiological agent can infect practically any organ or tissue in the body, causing multiple and diverse clinical manifestations^2^. The transmission occurs mainly during sexual contact; therefore, it is considered a sexually transmitted infection (STI)^3^. Transmission occurs at a higher rate owing to the fact that most infected people are unaware of their serological state because individuals may have few or no symptoms and can still transmit the disease to sexual partners^4^. The infection can also be transmitted through non-sexual contact, especially by blood transfusion or through the placenta during pregnancy (congenital syphilis)^5^. 

In 2016, the World Health Organization (WHO) estimated a prevalence of 19.9 million cases of syphilis in people aged 15-49 years, with an incidence of 6.3 million cases^6^. Noteworthy, this number may be underestimated because the prevalence of syphilis in the general population is better known in high-income countries^7^. In this sense, the WHO recommended strategies to combat STIs during 2016-2021, with a focus on reducing the global incidence of syphilis by 90% by 2030 and the cases of congenital syphilis per 100,000 live births by 50% in 80% of the affected countries^8^.

The Brazilian Ministry of Health reported 152,915 cases of acquired syphilis in 2019, representing an exceedingly high rate of 72.8 cases per 100 thousand inhabitants^9^. Regarding congenital syphilis, an incidence rate of 8.2 per 1,000 infants born alive was reported in 2019, a considerably higher number than the incidence rate reported in 2016, which was approximately 7.4 cases per 1,000 infants born alive^9^. 

Assessing and understanding the burden and trends of syphilis is essential for tracking control programs, especially considering the highly heterogeneous distribution of the disease in the Brazilian territory^10^. The epidemiological dynamics of syphilis have been addressed over the years in several studies. It is understood that various forms of syphilis in Brazil have been quantified at the national level and that differences have been found among regions, which justifies the need for performing subnational analyses. Analysis of the syphilis burden means looking far beyond information on the incidence and mortality of the disease. Additionally, a study on the disease burden could be an additional tool for understanding this serious health problem. To the best of our knowledge, this is the first study to analyze the syphilis burden in Brazil and its 27 federative units, using data from the Global Burden of Disease (GBD) Study from 1990 to 2019. We described the main disease burden metrics, incidence, deaths, years of life lost (YLLs), years lived with disability (YLDs), and disability-adjusted life years (DALYs). This research aimed to present the main burden imposed by syphilis generated by the GBD Study 2019 for Brazil and its 27 federated units.

## METHODS

### Study design and burden metrics data

A descriptive study was conducted using the metrics generated by the GBD Study 2019 for syphilis in Brazil and its 27 federated units from 1990 to 2019. The data for conducting the analyses were collected from the GBD electronic platform in May 2021^11^. The GBD is a project conducted by the Institute of Metrics and Health Assessment to create an extended and updated roadmap for health problems worldwide. The general methodological approaches used by the GBD Study 2019 to estimate these metrics have been published in previous studies^12^ ^-^ ^14^. 

### Case Definition

Among the “Communicable, maternal, neonatal, and nutritional diseases,” syphilis is classified under the topic “A.1.2 Sexually transmitted infections excluding HIV” in the GBD Study 2019 platform^12^ ^-^ ^14^. The causes of diseases were defined and identified according to the revisions of the International Classification of Diseases, 9^th^ (ICD-9) and 10^th^ (ICD-10) revisions. Specific definitions of ICD and modeling strategies for the cause of syphilis have been reported in other studies, with the ICD-10 codes A50-A53, ICD-9 codes 090-097, and the ICD-9 BTC code B060^12^ ^-^ ^14^.

### Data sources

The GBD data sources for Brazil have been previously described^12^ ^-^ ^14^. GBD mortality data, as well as the generation of YLL estimates, in Brazil are taken from the Mortality Information System adjusted by other national and international sources. The main sources of morbidity data, such as estimates for the YLD, are the Notification Information System for Notifiable Diseases, the Hospital Information System of the Unified Health System, and the Outpatient Information System of the Unified Health System. The GBD also considers publications in the scientific literature that highlight the prevalence of diseases and databases of Brazilian control programs when generating its estimates^15^ ^,^ ^16^.

For GBD, each death is attributed to a single underlying cause - the cause that initiated the series of events that had eventually led to death, according to the principles of the ICD. In the GBD Study 2019, data corrections were made for mortality underreporting and garbage redistribution codes for defined causes based on redistribution algorithms. Garbage codes are used to assign causes of death that cannot or should not be classified as the underlying cause of death. The GBD Study 2019 used the cause-of-death set model (CODEm), negative binomial regression, and natural history models to estimate the number of deaths due to syphilis by location, age group, gender, and year^12^ ^-^ ^14^. The GBD Study 2019 modeling strategy for morbidity estimation and validation has been published in previous studies, as well as the methods used to estimate the presented metrics^12^ ^-^ ^14^.

### Data assessment

Syphilis burden was assessed by determining the following metrics: incidence, death, YLLs, YLDs, and disability-adjusted life-years (DALYs = YLLs + YLDs). YLLs express the effect of premature deaths in a population and result from the multiplication of the number of deaths from syphilis in each age group by the standard life expectancy in that age group. For the GBD Study 2019, the standard life expectancy at birth was 67.2 years [95% uncertainty interval (UI) 66.9-67.6] in 1990, and 75.8 years (95% UI 75.4-76.2) in 2019 for Brazil^11^. YLDs express the sum of the prevalence of syphilis-related sequelae multiplied by the weight of the deficiency. Disability weighs the severity of health loss associated with the respective illness and is presented on a scale ranging from 0 (“perfect health”) to 1 (“equivalent to death”). YLLs and YLDs together provides data of DALYs. Estimates were presented as age-standardized rates per 100,000 inhabitants^12^ ^-^ ^14^. For characterization by age group, we analyzed the data of metrics in Brazil, considering the disease burden in people aged ≥10 years.

### Study area and time period

The Federative Republic of Brazil, located on the South American continent, ranks fifth considering the territorial extension, accounting for 8,510,345,538 km^2^. In terms of population size, it is the sixth largest country, with 211 million inhabitants as of 2020 and a population density of 22.43 inhabitants/km^2^ according to the 2010 census. Brazil is divided into 27 federated units. These 27 federated units form five geographic regions: North, Northeast, Midwest, Southeast, and South^17^. Regarding time period, we analyzed all metrics with their respective 95% UIs recorded in 1990, 2005, and 2019. The distribution maps of DALYs, YLLs, and YLDs metrics for the federated units were organized for the years 1990, 2005, and 2019 per 100,000 inhabitants and to relative percentages of change between the years (1990-2005 and 2005-2019), using QGIS 3.14 software (Norden, Germany). The shapefile was obtained from the platform of the Regional and Urban Economy Center of the State of São Paulo^18^. 

### Ethical aspects

This study was approved by the Research Ethics Committee of the Federal *Universidade Federal de Minas Gerais* (Project no. CAAE 62803316.7.0000.5149).

## RESULTS

### Syphilis burden in Brazil

Table 1 shows the rates per 100,000 inhabitants for syphilis in Brazil in 1990, 2005, and 2019. Concerning the three years compared, the incidence of the disease in the country was higher in 2019, with 136.7 (95% UI 106.7-171.0) cases per 100,000 inhabitants; this number showed an increase of 97.8% when compared with that in 2005. Most deaths were identified in 1990, with a rate of 0.3 (95% UI 0.2-0.4) per 100,000 inhabitants, with a percentage reduction (-66.7%) when compared with the data in 2005. Regarding disease burden metrics, there was a reduction in all rates per 100,000 inhabitants from 1990 to 2005: YLLs decreased by 58.8%; YLDs, by 25.0%; and DALYs, by 58.3%. Comparing 2005 and 2019, all metrics showed an increase: YLLs increased by 18.0%; YLDs, by 33.3; and DALYs, by 18.5%. Comparing 1990 and 2019, the incidence rate of syphilis in Brazil increased by 16.7% (Table 1). 

**TABLE 1:** Age-standardized rates of incidence, deaths, years of life lost due to premature death (YLLs), years lived with disability (YLDs), and disability-adjusted life years (DALYs) per 100,000 inhabitants and relative change, for syphilis in Brazil in 1990, 2005, and 2019. GBD Study 2019.

Metrics	Rate per 100,000 inhabitants (95% UI)			Relative change (%)		
	1990	2005	2019	1990 × 2005	2005 × 2019	1990 × 2019
**Incidence**	117.1	69.1	136.7	-40.9	97.8	16.7
	(87.0-156.3)	(53.5-87.9)	(106.7-171.0)			
**Deaths**	0.3	0.1	0.1	-66.7	0.0	-66.7
	(0.2-0.4)	(0.09-0.2)	(0.09-0.2)			
**YLLs**	25.5	10.5	12.4	-58.8	18.0	-51.3
	(20.6-32.0)	(8.8-12.4)	(9.8-15.3)			
**YLDs**	0.4	0.3	0.4	-25.0	33.3	0.0
	(0.2-0.5)	(0.2-0.4)	(0.2-0.6)			
**DALYs**	25.9	10.8	12.8	-58.3	18.5	-50.7
	(21.0-32.5)	(9.0-12.7)	(10.2-15.8)			

**95% UI:** 95% uncertainty interval, **YLLs:** years of life lost due to premature death, **YLDs:** years lived with disability, **DALYs:** disability-adjusted life years.

### Burden of syphilis in Brazil by gender

**FIGURE 1:**
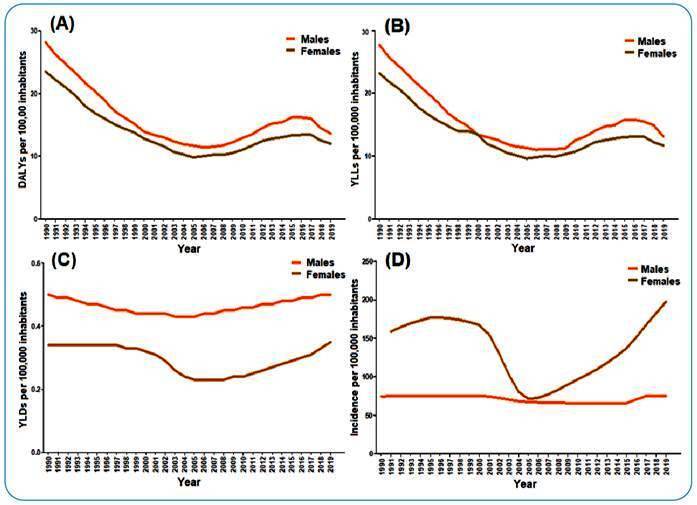
Age-standardized rates of metrics for syphilis by gender in Brazil, in the years 1990, 2005, and 2019. GBD Study 2019. **(A)** DALYs per 100,000 inhabitants. **(B)** YLLs per 100,000 inhabitants. **(C)** YLDs per 100,000 inhabitants. **(D)** Incidence per 100,000 inhabitants. **GBD:** Global Burden of Disease; **YLLs:** years of life lost due to premature death; **YLDs:** years lived with disability; **DALYs:** disability-adjusted life years.

Over the three years compared, the highest rates per 100,000 inhabitants of YLLs and DALYs were found in 1990 for males [YLLs: 27.7 (95% UI 22.9-35.6); DALYs: 28.2 (95% UI 22.7-36.0)] and females [YLLs: 23.2 (95% UI 18.4-29.5); DALYs: 23.5 (95% UI 18.8-30.0)] (Figure 1A and 1B ). The highest rates of YLDs, in turn, were verified for 1990 and 2019 for both sexes [males in 1990: 0.5 (95% UI 0.3-0.6); males in 2019: 0.5 (95% UI 0.3-0.6); females in 1990: 0.3 (95% UI 0.2-0.5); females in 2019: 0.3 (95% UI 0.2-0.6)] (Figure 1C). Males and females had reduced percentage change rates for all syphilis burden metrics when comparing 1990 and 2005 [YLLs (males: -59.2%; females: -58.6%); YLDs (males: -20.0%; females: -33.3%); and DALYs (males: -58.5%; females: -58.2%)]. Comparing 2005 and 2019, there was an increase in the percentage of changes for all disease burden metrics [YLLs (males: 15.9%; females: 21.8%); YLDs (males: 25.0%; females: 50.0%); and DALYs (males: 16.2%; females: 22.4%)] (Figure 1A, 1B, and 1C ). Males and females had incidence rates for syphilis per 100,000 inhabitants in Brazil, above 60 in the three years compared, but females had the highest values for the indicator during the study period (Figure 1D).

### Syphilis burden in Brazil by age range

**FIGURE 2:**
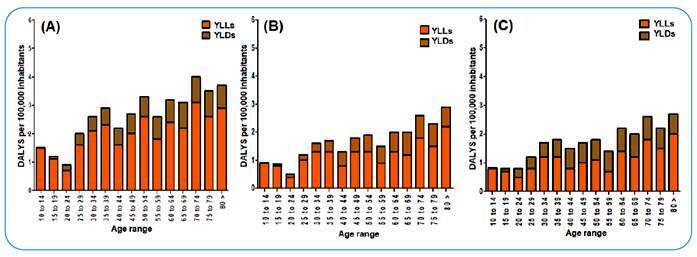
Age-standardized rates of metrics for syphilis by age range in Brazil. GBD Study 2019. **(A)** DALYs per 100,000 inhabitants in 1990. **(B)** DALYs per 100,000 inhabitants in 2005. **(C)** DALYs per 100,000 inhabitants in 2019. **GBD:** Global Burden of Disease; **YLLs:** years of life lost due to premature death; **YLDs:** years lived with disability; **DALYs:** disability-adjusted life years. 80 > = 80 years or more. *We plotted the data of these metrics from the GBD Study 2019 in Brazil considering the disease burden in people aged 10 years and above.

DALY rates per 100,000 inhabitants per age group showed the same distribution pattern in different periods. In 1990, 2005, and 2019, the highest DALY rates per 100,000 inhabitants occurred in patients above 50 years old, with variations in these rates between age groups and the highest DALY rate being in the 70-74 age group (Figures 2A, 2B, and 2C ). 

### Syphilis burden in Brazil by federated units

Regarding the highest rates of DALYs per 100,000 inhabitants among the years analyzed, Maranhão (Northeast region) ranked first in the three years. Pernambuco (Northeast region) ranked second in the incidence of syphilis in 1990, while Amapá (Northeast region) ranked the same position in 2005 and 2019. Piauí (Northeast region) ranked third in all the three years (Table 2).

**TABLE 2:** Age-standardized rates of disability-adjusted life years (DALYs) per 100,000 inhabitants for syphilis in Brazil, considering the Brazilian federated units, in 1990, 2005, and 2019. GBD Study 2019.

	Year					
Federated units	1990		2005		2019	
	DALYs (95% UI)	Ranking	DALYs (95% UI)	Ranking	DALYs (95% UI)	Ranking
**Maranhão**	165.2	1^st^	43.8	1^st^	29.2	1^st^
	(96.2-264.4)		(30.3-62.4)		(19.8-41.1)	
**Pernambuco**	47.3	2^nd^	20.8	2^nd^	22.4	4^th^
	(33.2-66.2)		(15.6-27.3)		(15.6-32.4)	
**Piauí**	46.0	3^rd^	20.1	3^rd^	22.7	3^rd^
	(29.1-69.2)		(14.0-29.0)		(13.1-37.9)	
**Tocantins**	34.3	4^th^	14.5	4^th^	15.6	8^th^
	(23.5-50.5)		(10.6-19.4)		(10.7-22.7)	
**Alagoas**	28.6	5^th^	11.2	8^th^	10.8	15^th^
	(19.4-43.0)		(8.5-14.8)		(7.3-15.9)	
**Rio Grande do Norte**	27.2	6^th^	10.2	10^th^	11.2	14^th^
	(18.9-39.5)		(7.6-14.2)		(6.9-16.9)	
**Espírito Santo**	25.4	7^th^	14.0	5^th^	17.0	6^th^
	(20.6-31.8)		(10.4-18.0)		(11.7-24.1)	
**Bahia**	24.5	8^th^	11.7	7^th^	16.6	7^th^
	(16.8-34.8)		(8.5-15.5)		(9.9-26.2)	
**Rio de Janeiro**	23.7	9^th^	11.0	9^th^	20.0	5^th^
	(19.2-29.2)		(8.7-13.8)		(15.2-25.7)	
**Ceará**	22.7	10^th^	8.5	13^th^	9.1	18^th^
	(12.7-38.3)		(6.0-11.8)		(5.6-14.2)	
**Amapá**	20.5	11^th^	12.7	6^th^	25.1	2^nd^
	(16.3-26.2)		(9.7-16.4)		(18.4-34.2)	
**Roraima**	20.0	12^th^	9.3	11^th^	15.1	9^th^
	(15.6-25.5)		(7.3-12.1)		(10.7-21.0)	
**Pará**	19.8	13^th^	9.1	12^th^	13.4	10^th^
	(14.8-26.6)		(7.0-11.9)		(8.4-21.1)	
**Paraíba**	19.6	14^th^	7.2	19^th^	6.6	23^rd^
	(13.5-28.6)		(5.4-9.6)		(4.5-9.3)	
**Acre**	15.4	15^th^	8.4	14^th^	11.7	13^th^
	(11.2-21.3)		(6.2-11.1)		(7.9-16.6)	
**Amazonas**	14.7	16^th^	11.7	20^th^	11.7	12^th^
	(11.2-19.2)		(8.5-15.7)		(8.5-15.7)	
**Mato Grosso do Sul**	14.6	17^th^	8.2	15^th^	9.4	17^th^
	(11.8-18.2)		(6.5-10.5)		(7.0-12.3)	
**Goiás**	14.6	17^th^	5.5	22^nd^	9.1	18^th^
	(11.3-18.4)		(4.3-7.1)		(6.7-12.4)	
**Minas Gerais**	14.4	18^th^	7.7	16^th^	9.7	16^th^
	(11.6-18.0)		(5.9-9.9)		(6.9-13.2)	
**Rio Grande do Sul**	13.8	19^th^	7.4	18^th^	12.4	11^th^
	(10.9-17.2)		(5.8-9.4)		(9.0-16.6)	
**Sergipe**	13.5	20^th^	7.6	17^th^	8.6	19^th^
	(9.9-18.8)		(5.6-10.0)		(6.2-11.7)	
**Rondônia**	12.9	21^st^	7.1	24^th^	7.1	21^st^
	(10.0-16.7)		(5.2-9.8)		(5.2-9.8)	
**Mato Grosso**	12.0	22^nd^	5.1	23^rd^	6.8	22^nd^
	(9.0-16.0)		(4.0-6.4)		(4.9-9.1)	
**Paraná**	11.9	23^rd^	6.6	20^th^	8.6	20^th^
	(9.5-14.9)		(5.0-9.0)		(5.7-12.2)	
**Santa Catarina**	9.4	24^th^	5.6	21^st^	7.1	21^st^
	(7.2-12.4)		(4.3-7.2)		(4.8-10.3)	
**Distrito Federal**	8.9	25^th^	4.2	25^th^	5.7	24^th^
	(6.9-11.3)		(3.3-5.2)		(4.1-7.7)	
**São Paulo**	7.3	26^th^	2.9	26^th^	5.4	25^th^
	(5.9-9.2)		(2.4-3.5)		(4.0-7.4)	

**95% UI:** 95% uncertainty interval; **DALYs:** disability-adjusted life years.

**FIGURE 3:**
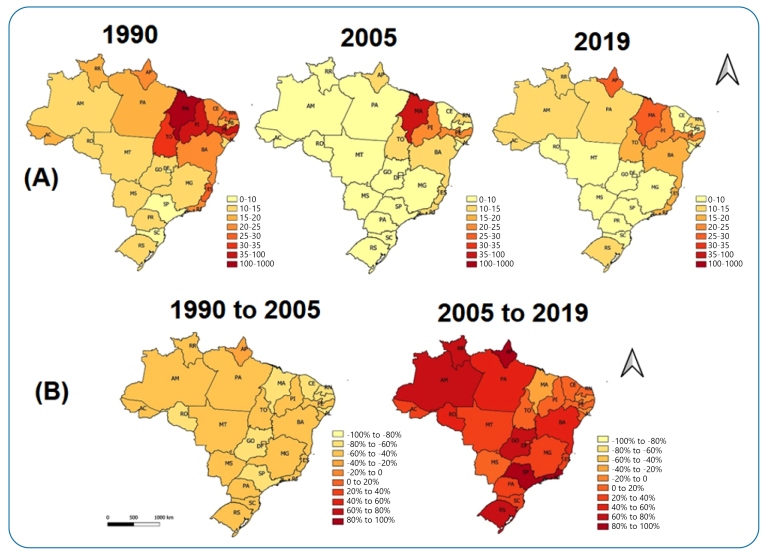
Standardized rates per 100,000 inhabitants in Brazilian federated units in 1990 and 2019 and percentages of change calculated for DALYs. **(A)** Rates for DALYs. **(B)** Percentage of change calculated for DALYs. GBD Study 2019. **GBD:** Global Burden of Disease; **DALYs:** disability-adjusted life years.

Among the Brazilian regions, the states of Tocantins (1990 and 2005) and Amapá (2019) (North region); Maranhão (1990, 2005, and 2019) (Northeast region); Mato Grosso do Sul (1990, 2005, and 2019) (Central-West region); Espírito Santo (1990 and 2005) and Rio de Janeiro (2019) (Southeast region); and Rio Grande do Sul (1990, 2005, and 2019) (South region) had the highest rates for DALYs per 100,000 inhabitants (Figure 3A). 

All federated units showed a decrease in the rates of DALYs per 100,000 inhabitants during 1990-2005 (Figure 3B). When comparing 2005 and 2019, there was an increase in the change percentage of the rates of the same metric in almost all federative units, except for the states of Alagoas, Maranhão, and Paraíba (Northeast region), which continued to have negative percentages (**Figure 3B**). The highest percentage increase was observed in the state of Amapá (North region), with a rate of 97.6% for DALYs.

## DISCUSSION

The analysis of syphilis burden metrics in the present study provides data on mortality and morbidity, which will enable the estimation of the impact of this disease on the health status of the Brazilian population. The results revealed a reduction in all syphilis indicators from 1990 to 2005 and a notable increase from 2005 to 2019. The most recent rate of DALYs for syphilis reported in 2019 revealed YLLs as the main contributor, which corresponded to 96.8% of its full value. Notably, the state of Maranhão (Northeast region) had the highest rates for syphilis burden metrics in all the three years provided, even though all Brazilian states had the increase observed when comparing 2005 and 2019. 

Despite decades of epidemiological and clinical experience with syphilis during pregnancy (asymptomatic or symptomatic women who are in the prenatal period, childbirth, and/or puerperium present at least one reactive or non-reactive treponemal with any titration)^19^ and congenital syphilis (stillborn, newborn, or abortion of a pregnancy in a woman with untreated or inadequately treated syphilis)^20^, both these conditions continue to remain as important public health problems in Brazil and the Americas^21^. In Brazil, congenital syphilis became a notifiable disease on December 22, 1986^22^. Nonetheless, syphilis in pregnant women was included in the national mandatory notification registry only on July 14, 2005^23^.

With these changes in syphilis records in the country, epidemiological surveillance of the disease indicated a growth in the rates of congenital syphilis. The incidence rate of congenital syphilis increased three times, from 2.4 to 8.2 per 1,000 live births, and the detection rate of syphilis in pregnant women increased six times, from 3.5 to 20.8 cases per 1,000 live births, when comparing 2010 and 2019^9^. In Brazil, controlling gestational syphilis is a great challenge, especially in the North and Northeast regions, where difficulties in controlling the disease persist^24^. To update interventions aimed at pregnant women, it is crucial to recognize the complex and dynamic determination of STIs, paying special attention to risk factors concerning sociodemographic, behavioral, and living conditions, and organizational changes in the health system and services^25^. The record of higher rates of maternal and congenital syphilis over the years may be attributed to the adoption of more effective measures by the epidemiological surveillance to identify cases and to the wide distribution and application of rapid syphilis tests^26^ ^,^ ^27^. Prenatal testing for syphilis reduced difficulties in patient follow-up while also revealing higher rates of syphilis in Brazil than expected^21^ ^,^ ^26^ ^,^ ^28^. Moreover, the shortage of benzathine penicillin G (BPG), procaine, and crystalline formulations, which are used for syphilis treatment, during 2014-2017 may have directly influenced the increase in the incidence of the disease in this population^27^.

Since 2010, the Brazilian Ministry of Health has adopted acquired syphilis as a case definition criterion for notification in the National List of Compulsory Notifications of Diseases, Illnesses, and Events in Public Health. This notification will be registered through the individual notification form of the Notification Information System for Notifiable Diseases. According to this system, an individual (non-pregnant female or male) with acquired syphilis is defined as being asymptomatic with a reactive non-treponemal test with any titration or reactive treponemal test, and without any record of previous treatment or being symptomatic with at least one treponemal reagent test, with any titration^20^.

Studies suggest that the increase in cases of acquired syphilis in Brazil is related to high-risk sexual behavior, coinciding with a new infection by human immunodeficiency virus^29^ ^-^ ^31^. In 2015, the detection rate of acquired syphilis was 42.7 cases per 100,000 inhabitants, with a ratio of cases between genders of 1.5 cases in males for each case in females, and 55.6% of the notifications were in individuals aged 20-39 years^32^ ^,^ ^33^. From 2010 to 2016, 227,663 patients were diagnosed with acquired syphilis^34^ ^,^ ^35^. Comparing 2010 and 2019, the detection rate of acquired syphilis increased 35 times, from 2.1 cases to 72.8 cases per 100,000 inhabitants^9^. Therefore, extensive information campaigns should be combined with preventive actions by the government and non-governmental organizations^36^. The Ministry of Health of Brazil, through the Unified Health System offers free testing and treatment in basic health units for syphilis, considering differentiated tests and therapeutic regimens for syphilis in pregnancy, congenital syphilis, and acquired syphilis^35^. 

As previously mentioned, syphilis is a disease for which the epidemiology is heterogeneous between sexes. Like other STIs, not only do men and women present different levels of biological risk of syphilis acquisition, but their clinical manifestations of infection and their behavioral likelihood of acquiring and transmitting syphilis are also distinct^37^. In this study, males presented higher rates of YLLs, YLDs, and DALYs per 100,000 inhabitants than females, indicating a greater disease burden among Brazilian men. Despite this, there has recently been a greater increase in these indicators in females when compared with that in males, which demonstrates the vulnerability of this population to the disease. 

A large difference in the incidence of the disease between the two sexes was noticeable. In the Brazilian scenario, it is known that men seek health care to a lesser extent^38^, which would explain the lower rates of early detection and, consequently, late diagnosis, leading to greater morbidity in this sex. However, women access health services more, but the Brazilian Ministry of Health also recommends screening for syphilis in the first and third trimesters of pregnancy^39^, ensuring an early diagnosis, with greater chances of cure and fewer complications, leading to a lower disease burden in this gender. The observed increase in syphilis detection in women can also be partially attributed to a change in the criteria for defining cases during pregnancy for surveillance purposes^9^. Unlike asymptomatic non-pregnant women or men, for whom two reactive tests are needed, namely, one non-treponemal and another treponemal test, the Brazilian Ministry of Health allows the diagnosis of syphilis during pregnancy with only one reactive test, making the diagnosis more sensitive in this population^9^.

The pattern of distribution rates of DALYs per 100,000 inhabitants was similar across all age groups in the three years. For all age groups, 1990 represented a year with the highest values of YLLs and YLDs (DALYs) and that these indicators demonstrated a reduction in 2005 and 2019. However, it was noted that after 25 years of age, regardless of the year, there were higher rates of DALYs, showing that adults and elderly people in Brazil have a greater syphilis burden. It is also known that these populations correspond to most notifications in the country. There was an increase in the detection rate for all age groups until 2018, with a subsequent reduction in 2019, highlighting the most accentuated trend of increase in the 20-29 years age group, which, in 2018, reached 165.4 cases per 100,000 inhabitants and, in 2019, was 163.0 cases per 100,000 inhabitants^9^. These data show the need for a more in-depth analysis of possible factors associated with YLLs lost owing to disability and premature death in older age groups compared with that in children and teenagers.

As previously described in the literature^10^, this study demonstrated that the Northeast region, particularly the state of Maranhão, is the place most affected by the disease and has higher disease burden indicators. The higher morbidity and mortality of the disease in this region are multifactorial, and some hypotheses explain this phenomenon. Among them, the smaller number of healthcare professionals and fragile health services in this region is apparent^40^, as well as failures in the use of diagnostic and treatment protocols by healthcare professionals^10^. Moreover, it has been previously shown that the majority of the maternal syphilis diagnoses in this state occurred at the time of childbirth^41^, a fact that indicates deficiencies in prenatal care held in the region and, consequently, leads to an increase in burden indicators. It is known that a lower level of education hinders people's access to information, generating a limited understanding of the importance of healthcare and, consequently, of preventive measures^40^. In Brazil, the region with the highest number of cases of acquired syphilis was precisely the region with the highest rate of illiteracy^42^, reinforcing the importance of health education as a preventive measure for the disease.

The data presented in this study have limitations regarding the coverage and quality of the databases used by the GBD Study 2019 and the inequalities between Brazilian federated units. It should be considered that the GBD Study 2019 estimates do not consider the classification of syphilis cases as adopted by the Brazilian Ministry of Health^20^, considering notifications of syphilis in pregnant women, congenital syphilis, and acquired syphilis separately. For the next estimates generated concerning this categorization, the GBD Study 2019 could estimate them separately, owing to different gender and age group aspects for the presentation of epidemiological data. However, it is noteworthy that the analysis of the GBD Study 2019 is very comprehensive and details on the syphilis burden in Brazil and its federated units and therefore represents a great improvement in the evidence for one of the most prevalent STIs and its impacts on population health. To the best of our knowledge, this is the first study to evaluate DALYs, YLLs, and YLDs in the context of syphilis in Brazil.

Assessing the syphilis burden allows for better interpretation of published data and observed regional variations, reinforcing the need for new policies adapted to the reality of each Brazilian region. Regional trends in DALYs caused by syphilis must be carefully analyzed to adopt control strategies specific to the realities of each region, thus reducing the disease burden on the country's public health. 

In conclusion, the results showed increasing syphilis burden. Males presented higher DALYs in the study years, although the incidence was higher in females. Syphilis affects many people of all age groups, especially those aged above 50 years, causing different degrees of disability and premature death (DALYs).
